# 5′-UTR G-Quadruplex-Mediated Translation Regulation in Eukaryotes: Current Understanding and Methodological Challenges

**DOI:** 10.3390/ijms26031187

**Published:** 2025-01-30

**Authors:** Polina N. Kamzeeva, Vera A. Alferova, Vladimir A. Korshun, Anna M. Varizhuk, Andrey V. Aralov

**Affiliations:** 1 Shemyakin-Ovchinnikov Institute of Bioorganic Chemistry, Miklukho-Maklaya 16/10, 117997 Moscow, Russia; 2 Lopukhin Federal Research and Clinical Center of Physical-Chemical Medicine, Federal Medical Biological Agency, 119435 Moscow, Russia; 3 RUDN University, 117198 Moscow, Russia

**Keywords:** RNA G-quadruplex, translation regulation, IRES, helicases, FMRP, 5′-UTR

## Abstract

RNA G-quadruplexes (rG4s) in 5′-UTRs represent complex regulatory elements capable of both inhibiting and activating mRNA translation through diverse mechanisms in eukaryotes. This review analyzes the evolution of our understanding of 5′-UTR rG4-mediated translation regulation, from early discoveries of simple translation inhibitors to the current recognition of their multifaceted regulatory roles. We discuss canonical and non-canonical rG4 structures, their interactions with regulatory proteins, including helicases and FMRP, and their function in both cap-dependent and IRES-mediated translation. Special attention is given to the synergistic effects between rG4s and upstream open reading frames (uORFs), stress-responsive translation regulation, and their role in repeat-associated non-AUG (RAN) translation linked to neurodegenerative diseases. We critically evaluate methodological challenges in the field, including limitations of current detection methods, reporter system artifacts, and the necessity to verify rG4 presence in endogenous transcripts. Recent technological advances, including genome editing and high-throughput sequencing approaches, have revealed that rG4 effects are more complex and context-dependent than initially thought. This review highlights the importance of developing more robust methodologies for studying rG4s at endogenous levels and carefully reevaluating previously identified targets, while emphasizing their potential as therapeutic targets in various diseases.

## 1. Introduction

RNA molecules exhibit remarkable structural versatility, orchestrating diverse processes, including translational modulation and subcellular localization, thereby facilitating rapid cellular adaptation to environmental changes [1,2]. The application of advanced structural biology methodologies, in conjunction with high-throughput sequencing technologies, has revealed that this regulatory capacity is derived from RNA’s exceptional structural plasticity and dynamic conformational landscapes [3]. The repertoire of structural motifs includes, but is not limited to, stem-loops, pseudoknots, triple helices, i-motifs, and G-quadruplexes.

RNA G-quadruplexes (rG4s) have been identified as a particularly significant regulatory element. These structures, preferentially formed in guanine-enriched sequences, are characterized by planar arrangements of four guanine residues (G-quartets) stabilized through Hoogsteen hydrogen bonding, with multiple G-quartets stacking to form the rG4 [4]. The structural integrity of these complexes is maintained through coordination with monovalent cations, particularly potassium, which occupy the central channel between stacked G-quartets. Although rG4s exhibit robust stability under physiological conditions in cuvette, the question of their intracellular formation remains unresolved. This necessitates the use of diverse analytical methodologies, including chemical probing, immunological detection, and high-throughput sequencing approaches, to achieve comprehensive characterization [3]. The results of mapping rG4s in transcripts using techniques based on reverse transcriptase stalling (rG4-seq) [5] or affinity capture coupled with sequencing (G4RP-seq) [6] suggest that rG4s are prevalent in eukaryotic cells, while DMS-seq [7] data suggest that rG4s are globally unfolded. The formation of rG4s in cells has been supported by ^19^F NMR spectroscopy [8], immunofluorescence microscopy with a G4-specific antibody BG4 [9], and real-time visualization using an rG4-specific fluorescent probe QUMA-1 [10]. These findings have led to the hypothesis of transient rG4 formation. According to this hypothesis, G-rich RNA sequences that adopt rG4 structures, hereafter referred to as “folded rG4s”, and unstructured G-rich sequences, hereafter referred to as “unfolded rG4s”, exist in a dynamic equilibrium that is subject to precise cellular regulation [11]. Strong evidence for the transient formation of rG4s in human cells has been provided by chemical crosslinking-based assays, which enable the global capture of rG4 snapshots [6]. Time-resolved analyses using this technique confirmed that G4s can switch between folded and unfolded states within seconds [12,13,14,15]. The equilibrium between unfolded rG4s, folded rG4s, and other possible RNA secondary structures (e.g., hairpins) depends on multiple factors, including the activity of rG4-resolving helicases, interactions with rG4-binding proteins, the presence of G4-ligands, potassium cation (K^+^) concentration, and molecular crowding [4,14,15].

Folded rG4s within 5′-UTRs tend to inhibit the progression of scanning pre-initiation complexes (PICs) and, therefore, act as translational repressors [4,16]. However, certain rG4s act as elements of internal ribosome entry sites, promoting cap-independent translation. The regulatory potential of 5′-UTR rG4s is diverse due to their complex interplay with upstream open reading frames or cis- and trans-regulatory elements, including rG4-interacting proteins [17,18,19,20]. Recent investigations have substantially expanded our comprehension of rG4 structural heterogeneity, physiological relevance, and intricate mechanisms governing translational control. This review aims to examine the mechanisms of rG4-mediated translational regulation, evaluate methodological developments, and update the inventory of potential rG4-containing 5′-UTR regulatory elements affecting translation efficiency.

## 2. Understanding the Specificity of rG4 Formation

The formation of both DNA and RNA G4s necessitates specific sequence requirements, namely the presence of four G-tracts interspersed with nucleotide loops. Traditional criteria for the stable formation of DNA G4s postulate that each G-tract must comprise three or more consecutive guanines, with intervening loops not exceeding seven nucleotides, conforming to the canonical motif G_3+_-N_1–7_-G_3+_-N_1–7_-G_3+_-N_1–7_-G_3+_ [21]. This canonical motif was extensively used in rG4 search [22]. However, neither G-rich content nor canonical motif compliance is sufficient to predict DNA and RNA G4 formation or functional regulatory capacity.

Multiple factors influence rG4 assembly, including sequence composition (G-tract number, loop length, and composition) and context, global mRNA secondary structure, and physiological conditions [23,24,25,26]. The presence of C-rich flanking regions can inhibit rG4 formation by promoting Watson–Crick base-pairing and the subsequent formation of alternative structures [24]. Additionally, broader mRNA folding patterns may favor alternative structural conformations, potentially precluding rG4 assembly [25]. Notably, K^+^ concentration serves as a critical modulator of rG4 stability, suggesting that physiological K^+^ fluctuations may dynamically regulate cellular rG4 landscapes [23].

The formation of rG4s exhibits a complex relationship with sequence motifs. Three-tetrad rG4 structures having loops up to 3 nt demonstrated similar thermodynamic stability, regardless of how the loop lengths were arranged, as long as the total loop length was preserved [26]. A 2013 study investigated how loop length and composition affect the thermodynamic stability of two- and three-tetrad rG4s [23]. Thermodynamic stability of three-tetrad rG4s demonstrated an inverse correlation with loop length until it reached the 9 nt threshold, after which the stability plateaued. Two-tetrad rG4s exhibiting loop lengths, each greater than 3 nt, display significantly reduced stability. Adenine was found to be favored over uridine and cytosine in loops of two-tetrad rG4s, presumably because the latter allow for alternative structures with Watson–Crick G-C and wobble G-U base pairing. Most importantly, this study emphasized the need to search for non-canonical two-tetrad and long-looped rG4s in naturally occurring RNAs. Non-canonical rG4s, including two-tetrad variants [27] and those containing bulges [28] or extended loops [29,30], represent a significant subset of rG4s in biologically relevant RNAs. Moreover, DHX36, a G4-unwinding helicase, was shown to predominantly target non-canonical rG4s containing two tetrads and bulges in the 5′-UTRs of myoblasts’ transcripts [31]. Non-canonical rG4s identification poses considerable challenges, as attempts to broaden general motif parameters typically result in elevated false-positive rates [22]. Current approaches employ multiple strategies, including diverse motif sets [32], machine learning algorithms [33,34], and G/C content analysis [33].

rG4s exhibit distinct structural preferences, predominantly adopting parallel topologies with uniform 5′-to-3′ strand orientation, contrasting with DNA G4 topological diversity [23,26,35]. This topological constraint stems from the presence of 2′-hydroxyls and the preference for guanosines in the anti-conformation. However, research on RNA aptamers demonstrates that non-canonical structural elements can facilitate alternative rG4 topologies, including antiparallel arrangements with strand polarity alterations and alternating syn-/anti-conformations of guanosine residues along the strand [36]. While high-resolution techniques such as X-ray crystallography and NMR spectroscopy, supplemented by small-angle X-ray scattering and dynamic light scattering analyses, have been primarily applied to aptamer studies [36], investigations of 5′-UTR rG4s typically rely on circular dichroism (CD) spectroscopy and biochemical probing techniques, albeit with reduced structural precision. The focus on predominantly canonical rG4s and the utilization of methods that preclude precise structural determination may account for the fact that, to date, only one putative antiparallel rG4 has been identified among naturally occurring 5′-UTR rG4s [37].

Sequence conservation provides compelling evidence for rG4 regulatory significance [5,38]. Comparative genomic analyses reveal substantial evolutionary preservation of 5′-UTR rG4-forming sequences across mammals, encompassing both structural features and positional conservation. Conservation patterns demonstrate a phylogenetic gradient, with the highest preservation among primates (75% for two-tetrad structures), reduced conservation in rodents (29%), and minimal presence in lower eukaryotes [38]. This evolutionary conservation pattern extends to numerous regulatory rG4s discussed in subsequent sections [31,39,40,41,42,43,44,45,46]. The marked evolutionary conservation of these elements, particularly in mammalian systems, coupled with their diverse regulatory functions, establishes rG4s in 5′-UTRs as critical modulators of translation. Since the predominant effect of 5′-UTR rG4s is translational suppression via impediment of PIC scanning, our analysis will commence with translation-inhibiting rG4s before advancing to more sophisticated mechanisms of translational enhancement.

## 3. Translation-Inhibiting rG4s

### 3.1. First Identified Translation-Inhibiting rG4s

First, translation-inhibiting rG4s in human mRNAs were found in the 5′-UTRs of NRAS and Zic-1 transcripts. For NRAS, the rG4’s influence on translation was demonstrated through in vitro translation assays using rabbit reticulocyte lysate [42]. The Zic-1 rG4 became the first target whose effect was demonstrated in cellulo using a dual luciferase reporter assay (DLR) coupled with RT-qPCR to distinguish between translational and transcriptional effects [43]. Further studies frequently used the same methods to evaluate the effect of rG4s in 5′-UTRs on translation efficiency.

**Figure 1:**
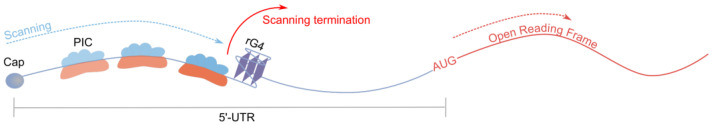
rG4 inhibits cap-dependent translation through steric hindrance.

The inhibitory effect of 5′-UTR rG4s on translation was initially attributed to steric hindrance created by the rG4 impeding mRNA scanning by PIC from the cap to the start codon (Figure 1). Model systems using DLR assay established a correlation between rG4 thermodynamic stability and translation inhibition efficiency [47,48]. However, no consensus was reached regarding how rG4 positioning within the 5′-UTR affects the magnitude of repression. Investigation of rG4-binding proteins’ influence on translation remained largely unexplored. A notable exception is Fragile X messenger ribonucleoprotein FMR1, which stabilizes rG4s and inhibits translation [49].

The discovery of regulatory rG4s in NRAS and Zic-1 5′-UTRs initiated a wave of reports between 2007 and 2012, identifying translation-inhibiting rG4s in the 5′-UTRs of biologically significant genes (Table 1). Most studies followed a common pipeline established in the pioneer publications: (1) bioinformatic identification of sequences in target mRNA 5′-UTRs with a potential to form rG4s; (2) investigation of the corresponding oligoribonucleotide structure using CD and UV spectroscopy, along with reverse transcriptase (RT) stop, RNase T1 and in-line assays; (3) comparison of rG4-forming sequence versus non-rG4-forming mutant effects on translation efficiency using reporter systems, primarily through DLR assay supplemented by RT-qPCR, less frequently via in vitro translation or Western blotting of transfected vector products. In some cases, researchers investigated the effects of G4 ligands on translation efficiency in reporter systems. For instance, 360A, Phen-DC3, and Phen-DC6 demonstrated a dose-dependent ability to decrease TRF2 translation efficiency, with reductions ranging from 15% to 90% [44], while TMPyP4 attenuated translation inhibition by approximately 40% for metalloproteinase MT3-MMP [50].

However, most studies of that period did not progress beyond reporter systems, and the downstream effects of rG4-controlled translation on biological processes remained unexplored. Only for CCND3 and ADAM10 was the impact of rG4 mutation on protein overexpression levels and intracellular processes demonstrated. Flow cytometry and MTT assays revealed that CCND3 overexpression by corresponding vector transfection under wild-type (WT) rG4 control only marginally stimulated cell S-phase transition and division, whereas the vector with non-rG4-forming mutant significantly increased both S-phase cell proportion and division rate [41]. For ADAM10, vectors with non-rG4-forming mutants showed elevated levels of APPsα, the primary protein activity product, in the supernatant [45].

Even in these cases, the effect was not confirmed at the endogenous mRNA level. Moreover, for rG4 in the 5′-UTR of Bcl-2, a known anti-tumor target and apoptosis inhibitor, CRISPR-Cas9-based deletion of the rG4-forming sequence from melanoma cell genome caused no changes in mRNA levels, protein levels, or response to apoptosis-activating compounds compared to unedited cells [52]. This led to the conclusion that at the endogenous mRNA level, the presence of rG4 does not significantly affect translation, and the effects observed in the reporter assays may result from putative oversaturation of the rG4-resolving helicase activity. This same issue is known for in vitro translation assays: for Bcl-2, no rG4-related inhibitory effect was observed at the 20 ng/μL plasmid concentration, but translation inhibition through rG4 became detectable (2-fold) at 320 ng/μL [39]. However, the study on Bcl-2 rG4 with CRISPR-Cas9 had limitations due to significant experimental error and the relatively weak effect of the chosen rG4. While this work undermines the prospects of Bcl-2 rG4 as an anti-tumor therapy target, it raised important questions about the adequacy of primary DLR and in vitro translation methods for assessing rG4’s influence on translation efficiency.

The mechanism of rG4-mediated translation inhibition remained largely unexplored, primarily reduced to the hypothesis of steric hindrance created by rG4s impeding scanning PIC. Except for rG4-resolving helicases and FMRP, rG4s protein partners were only identified for MT3-MMP, ARPC2, Bcl-2, and NRAS mRNAs [17,19,20]. The observed interactions demonstrated specificity for folded rG4s, as evidenced by the absence of binding with G-rich control sequences lacking rG4-forming capability, though the possibility remains that these proteins may exhibit binding affinity for additional molecular targets. Among these proteins were small ribosomal subunit proteins, generating a hypothesis about PIC scanning deceleration due to its proteins’ affinity for 5′-UTR rG4s.

In most cases of that period (except estrogen receptor 1 mRNA investigation), the critical step was overlooked, namely verifying expression levels of mRNA containing rG4-forming sequences and their transcription start sites [51]. It is well established that determining mRNA 5′-ends presents significant challenges, and mRNA sequence databases undergo constant refinement as methodology improves [56,57]. Furthermore, cell line-specific and condition-dependent variations in promoter activation and splicing patterns can alter target mRNA levels. Nevertheless, only for estrogen receptor 1 (ESR1) was the presence of rG4-forming sequences in cellular mRNAs confirmed by qRT-PCR, albeit with caveats regarding method sensitivity [51]. Surprisingly, for NRAS rG4, which served as a control in numerous studies, a near-complete absence of mRNAs containing the cap-proximal rG4-forming sequence was demonstrated across 14 cell lines [58]. According to an updated NCBI annotation, NRAS mRNA contains a 131 nt-long 5′-UTR, while the rG4 was previously reported 222 nt upstream of the translation start site (TSS). The conducted experiments, including qRT-PCR across 14 cell lines, rapid amplification of cDNA 5′-ends (5′-RACE) on HEK293T cells, and cap analysis of gene expression (CAGE)-Seq data confirmed that most NRAS mRNAs lack the rG4-forming sequence. In 2016, new rG4s were discovered in NRAS 5′-UTR using the rG4-specific ligand RGB-1; however, these rG4 sequences are also absent in the current annotation [59]. Thus, critical revision of other known rG4 targets is recommended for each tissue type/cell line since these structures might not actually be present in cellular mRNAs.

**Figure 2:**
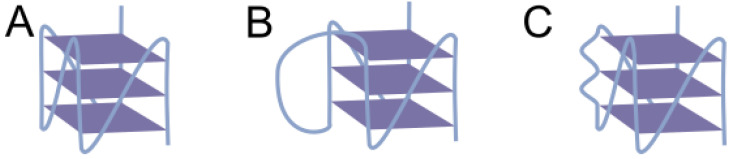
Canonical (**A**) and non-canonical rG4s with long loop (**B**) and bulges (**C**).

Additionally, many 5′-UTR rG4-forming sequence identifications may have been erroneous or incomplete, as contemporary G4 predictors relied on canonical motifs, while many rG4s possess non-canonical structures with long loops, bulges, two G-tetrads, etc. (Figure 2) [60]. Therefore, the investigation of most rG4 targets subsequently evolved toward (1) searching for non-canonical rG4s; (2) identifying rG4s whose biological role was supported by genome editing and helicase inhibition experiments; (3) discovering new mechanisms of rG4-mediated translation regulation in 5′-UTRs.

### 3.2. Non-Canonical rG4s Inhibiting Translation

In previously discussed examples, rG4s were mostly identified using the canonical motif (Figure 2A). Later, three-tetrad RNA and DNA G4s with loop lengths up to 15 nt and 30 nt, respectively, were shown to be stable at the physiological temperature in cuvette (Figure 2B) [23,61]. J.P. Perreault’s group was among the first to address this, conducting bioinformatic searches in 5′-UTR databases for rG4-forming motifs with two single-nucleotide loops and one long central loop up to 90 nt. They successfully identified rG4s inhibiting translation of therapeutically significant HIRA, TOM1L2, and APC mRNAs by 90%, 75%, and 40%, respectively, according to DLR assay data [29]. Analysis via in-line probing methodology revealed that these rG4 structures possessed loop regions spanning from 11 to 32 nt, whereby the assay provided detailed insights into the specific nucleotide contributions to structural assembly. In a similar study, 5′-UTRs of GRIA1, BNIP1, and TEF mRNAs were discovered to contain rG4s, each having one long peripheral loop from 15 to 26 nt [30]. According to DLR assay results, they also inhibited the translation of the corresponding mRNAs by 18%, 69%, and 81%, respectively.

Since the study revealed that G4s with loops longer than 30 nucleotides do not form regulatory sequences in cells (although they fold in cuvette), subsequent work employed a modified motif G_3_-N_1_-G_3_-N_1–20_-G_3_-N_1_-G_3_ [29,62]. This approach, combined with the canonical motif, identified three additional rG4 targets involved in intestinal cancer development that inhibit cap-dependent translation by 2–4 fold: BAG-1, MAPK3, and CASP8AP2. Analysis of in-line cleavage patterns revealed that G-rich sequences are able to form rG4s with loops longer than conventional 7 nt. Thus, new searching motifs and in-line probing expanded the repertoire of known rG4 regulators and identified several targets for anti-tumor therapy.

Non-canonical rG4s were also discovered during the investigation of the effects of G4 stabilizer RGB-1 on oncogene mRNA translation efficiency [63]. To identify targets whose translation, but not transcription, depends on RGB-1, Western blotting and qRT-PCR data were compared before and after RGB-1 cell treatment. This approach identified two targets, Nectin-4 and CapG. Nectin-4 regulates Ca^2+^-independent cellular adhesion and cancer proliferation [64]. CapG regulates NF-κB and PI3K/AKT signaling, enhances cell motility, and accelerates proliferation in cancers [65,66,67,68]. Although canonical rG4 motifs were present in the 5′-UTRs of both mRNAs’, RT-stop assay revealed rG4 formation in both cases to ignore the fourth adjacent G-tract, instead incorporating a G-tract located more than 7 nt away. Moreover, for CapG, the equilibrium between canonical/non-canonical rG4s was shown to depend on potassium concentration. At a near-physiological potassium concentration (100 mM), a non-canonical rG4 formed that can inhibit translation by 50%. At a lower potassium concentration (25 mM), rG4 formation results in only 20% translation inhibition. The authors suggested that this regulating mechanism of the translation efficiency in response to changes in potassium concentrations might have biological significance.

In addition to RT-stop and in-line assays, which identify residues forming rG4s, DLR assay can track translation inhibition efficiency depending on sequence mutations. This approach helped to establish the structural ensemble of highly polymorphic rG4s in the 5′-UTR of mRNA coding VWF, an antihemophilic factor carrier, and platelet-vessel wall mediator, whose deficiency leads to von Willebrand disease [69,70]. Its 5′-UTR contains three GGG tracts, flanked by several GG tracts and isolated Gs at both 5′- and 3′-ends. When G-to-A mutations were introduced into the G-rich sequence at the 5′-end, the inhibitory effect on translation increased compared to WT rG4, suggesting the formation of a more homogeneous and stable structure by the remaining guanines and indicating the minimal contribution of 5′-terminal guanines to the major inhibitory structure. Conversely, mutation of most guanines at 3′-end attenuated the inhibitory effect, likely due to rG4 structure destabilization. Analysis revealed that G38 and G45 likely participate in the formation of most ensemble structures, while the intervening G residue between them can vary (although G42 is predominantly involved), with bulges forming in all cases (Figure 2C). While this system clearly exceeds the resolution of employed methods and requires confirmation by NMR or X-ray crystallography, this study raises questions about expanding bioinformatic rG4 search motifs to include bulge-containing patterns. Thus, the approach expanded the repertoire of known rG4 translation inhibitors.

### 3.3. Involvement of rG4-Binding Proteins in Translation Inhibition

#### 3.3.1. Investigation of Inhibitory rG4s in Context of rG4-Resolving Helicase Interaction

**Figure 3:**
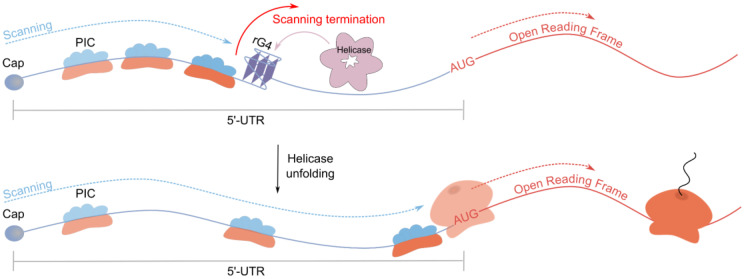
A rG4-resolving helicase alleviates rG4-dependent translation inhibition.

As the field developed, it became possible to study rG4’s influence on translation in the context of rG4-binding protein interactions. DHX36 (also known as RHAU, G4R1) is of particular interest as a helicase with high folded G4 affinity and predominantly cytoplasmic localization, capable of unwinding rG4s and thereby regulating translation, though this helicase can also bind non-rG4-forming AU-rich sequences through another binding site (Figure 3) [71]. DHX36 plays a special role during active cell proliferation, including embryonic development and injury recovery. Consequently, DHX36 expression decreases during embryonic and stem cell differentiation [6,31,40]. In myoblasts, DHX36 targets, most of which contain rG4-forming motifs, were shown to have the highest density in 5′-UTRs, and the helicase knockdown primarily decreased the translation efficiency of mRNAs containing 5′-UTR rG4s [31]. Thus, DHX36 can enhance the translation efficiency of rG4-containing mRNAs in rapidly proliferating cells, and its knockdown/overexpression can serve as a tool for identifying rG4 targets, including non-canonical ones.

Mice with conditional DHX36 knockout in myogenic cells showed smaller fibers, limb muscle weakness, and significantly reduced regeneration efficiency after injury, attributed to a decreased rate of cell proliferation [31]. Search for downstream targets of DHX36 knockout whose translation decreases without mRNA level changes in myoblasts using cross-linking and immunoprecipitation (CLIP)-seq and polysome profiling coupled with RNA-seq identified mRNA encoding G protein subunit alpha i2 (GNAI2). This protein participates in the hormonal regulation of adenylate cyclase and regulates cell proliferation, muscle size, and regeneration. The GNAI2 5′-UTR mRNA contains a G-rich conservative sequence where two rG4-forming sequences were found by probing with fluorogenic dyes NMM and ThT along with RT-stop assay. RNA pull-down revealed the 5′-UTR association with DHX36, and the anti-DHX36 antibody effectively retrieved GNAI2 mRNA via RNA immunoprecipitation (RIP). GNAI2 protein levels were significantly reduced without mRNA level changes in DHX36-knockout stem cells and myoblasts, with effects enhanced by the treatment with rG4-stabilizer cPDS, alongside a decreased proliferation rate. Insertion of full-length 5′-UTR and rG4-forming sequences into a plasmid vector upstream of EGFP-coding sequence downregulated translation compared to non-rG4-forming mutants according to Western blotting data. The inhibitory effect of the rG4s on the vector translation was more pronounced in DHX36 knockout cells. Moreover, DHX36 plasmid overexpression increased GNAI2 protein levels and rescued stem cell proliferation by 30.8%. Therefore, GNA2I rG4s can be considered promising targets, with the potential for improving injured muscle recovery efficiency.

DHX36 deletion also disrupts heart development in mouse embryos, leading to death at day E7.5 due to cardiomyocyte proliferation impairment [40]. RNA microarray analysis, Northern blotting, qRT-PCR, and whole-mount in situ hybridization (WISH) revealed increased levels of Nkx2-5 mRNA. However, protein levels, according to Western blotting analysis and immunofluorescence (IF) staining, decreased with DHX36 knockout. Investigation of this phenomenon using RIP and RNA pull-down assays showed that DHX36 could form complexes with both 5′- and 3′-UTRs of Nkx2-5 mRNA. When binding to 3′-UTR, DHX36 accelerates mRNA degradation; hence, DHX36 deletion leads to increased mRNA levels. The Nkx2-5 5′-UTR mRNA contains a highly conserved G-rich sequence capable of forming rG4. The Nkx2-5 5′-UTR sequence, when inserted into a GFP expression vector, inhibited its translation. In the presence of DHX36, the GFP levels significantly increased, indicating improved translation efficiency due to the unfolding of the inhibitory rG4. Inserting this 5′-UTR with the WT rG4 and corresponding 6G-A mutant into an Nkx2-5 expression vector increased translation efficiency according to Western blotting data. DLR assay showed that DHX36 knockdown reduced the translation efficiency of Nkx2-5 5′-UTR containing mRNA by 49%. An addition of siDHX36 also decreased the proportion of the mRNA in the polysomal fraction. Notably, Nkx2-5 rG4 contains five G-tracts and must include a 15-nucleotide loop in any assembly, indicating its non-canonical structure.

Based on rG4-seq data, an rG4 target was found in the 5′-UTR of ADAR1 mRNA [5]. ADAR catalyzes adenosine deamination to inosine in RNA substrates, thereby modulating splicing, translation, and RNA stability [72,73]. RT-stop and selective hydroxyl acylation analyzed by lithium-ion mediated primer extension (SHALiPE) assays were used to precisely determine nucleotides participating in the structure formation [74]. In the presence of DHX36, the rG4-forming oligoribonucleotide showed a greater tendency to form duplexes with a complementary trap than intramolecular rG4, according to native electrophoresis data. The ability to form rG4 in cellulo was supported by confocal microscopy: upon ADAR 5′-UTR RNA transfection, cell fixation, and permeabilization, colocalization was observed between FAM-adar and ISCH-adar oligonucleotide probes complementary to ADAR rG4 flanking sequences and fluorescently labeled with FAM and G4-specific light-up moiety, respectively. When repeating the experiment in DHX36-knockout cells, the rG4 light-up/FAM signal ratio increased 2-fold compared to intact cells, indicating increased stable rG4 proportion. According to DLR assay, rG4 separately and in the context of the whole ADAR 5′-UTR reduced the translation efficiency of the reporter by 2.7-fold and 4.3-fold, respectively, compared to non-rG4-forming mutants. In the former case, transfection of cells with WT rG4-containing plasmid followed by the treatment with G4 stabilizer PDS led to an additional 3.0-fold decrease in translation efficiency compared to DMSO. When conducting the DLR assay under DHX36 knockdown and knockout conditions, the translation efficiency of WT rG4-bearing mRNA was decreased additionally by 1.6- and 2.9-fold compared to intact DHX36 levels. The rG4’s influence on translation was also confirmed by Western blotting using vectors containing ADAR1 5′-UTR, and the coding sequence (CDS), with translation efficiency decreasing in DHX36 knockout cells. Thus, the ADAR1 rG4 study demonstrated DHX36 level influence on stable rG4 proportion.

The 5′-UTR mRNA of the TWIK-related acid-sensitive K+ channel (Task3) was also shown to have a regulatory rG4 [75]. Task3 dysfunction leads to impaired neuronal migration of cerebral cortical neurons in developing murine brains and maternally imprinted intellectual disability Birk-Barel mental retardation. The repeat (GGN)_13_, due to its length and polymorphic nature, represented a complex object for investigation, including control selection. Therefore, the authors proposed using GC-scrambled or complementary sequences as controls, along with comparing CD spectra before and after DMS treatment. RIP with G4-binding BG4 antibody confirmed intracellular Task3 rG4 formation. Western-blotting of 3xFLAG-tagged products of reporter constructs possessing WT or non-rG4-forming mutant regions upstream of Task-M1 CDS revealed the translation inhibition for WT construct by 3-fold compared to the reporter containing a GC-scrambled sequence of identical length. FLAG immunoprecipitation experiments validated the molecular interaction between DHX36 and Task3 mRNA, as evidenced by the transfection of a construct encoding N-terminally FLAG-tagged full-length DHX36. DHX36 overexpression increased the translation efficiency of the Task3-M1 coding reporter construct by 2-fold and endogenous Task3 expression by 1.25-fold, increasing cell hyperpolarization in the second case. Thus, Task3 5′-UTR rG4, which mediates Task3 production affecting cell hyperpolarization and whose existence in endogenous mRNA was confirmed by 5′-RACE, represents an attractive target for developing therapeutic agents against Birk-Barel mental retardation.

Recently, an rG4 was identified in 5′-UTR of PBX1 mRNA [76]. PBX1 is a transcription factor whose overexpression is linked to pre-B-cell acute lymphoblastic leukemia and melanoma development. Using a GFP-expressing reporter construct, PBX1 non-rG4-forming mutation was shown to improve translation efficiency by approximately 20%. Furthermore, G4 ligands PDS and TMPyP4 inhibited translation to 25% of the WT level. Additionally, helicase DHX9 was demonstrated to regulate PBX1 mRNA translation by resolving the rG4 structure. PBX1-rG4 edited cell lines showed no dependence on rG4-stabilizing compounds, and immunohistochemistry of normal and tumor tissues revealed that DHX9 absence corresponded to PBX1 absence. Notably, DHX9 knockdown in melanoma reduced tumor progression.

In the hepatitis B virus (HBV)-related hepatocellular carcinoma context, RNA helicase DDX5 was known to be downregulated during HBV replication, correlating with poor prognosis. Recent research has demonstrated that DDX5 facilitates STAT1 transcription factor translation; specifically, siRNA-mediated DDX5 knockdown reduces STAT1 protein levels without affecting STAT1 mRNA levels or splicing. Transcriptome-wide rG4-seq revealed that the 5′-UTR of STAT1 mRNA exhibits significant potential for rG4 formation [5]. Three G-rich sequences potentially capable of forming rG4 were discovered in STAT1 5′-UTR mRNA, with the cap-proximal sequence’s influence on translation thoroughly investigated [77]. According to the DLR results, the mutation of the cap-proximal rG4 increased the translation efficiency by more than 2-fold. Simultaneously, both the treatment with rG4 stabilizer (Phen-DC3 or PDS) and DDX5 knockdown decreased the translation efficiency in the reporter system without significantly affecting the non-rG4-forming mutated vector. This indicated rG4’s inhibitory effect on STAT1 mRNA translation, whose degree depends on DDX5 helicase activity. To obtain data about the effects on endogenous mRNA levels, CRISPR-Cas9 was used to delete the rG4-forming sequence from the STAT1 gene. Cells with biallelic deletion showed a significant and reproducible increase in STAT1 protein levels compared to unedited cells, while STAT1 mRNA levels remained unchanged. DDX5 knockdown and treatment with Phen-DC3 and PDS had no effect on STAT1 protein levels in biallelic cells but reduced them in WT cells. DDX5 and STAT1 mRNA rG4 interaction was also confirmed by RIP, RNA pull-down, and electrophoretic mobility shift assay (EMSA) methods. The study also showed that HBV replication reduces DDX5 levels and STAT1 levels, clinically significant for interferon therapy. In addition to therapeutic significance, this case provided the first example of rG4, whose influence at endogenous mRNA level was confirmed by genome editing methods, and drew attention to helicase DDX5, which was less known than DHX36 and DHX9.

Thus, helicases DHX36, DHX9, and DDX5 significantly regulate the degree of rG4-mediated translation inhibition. This is important from two perspectives. First, it opens therapeutic horizons for controlling rG4-regulated mRNA translation through helicase activity and, conversely, for increasing translation efficiency by G4-specific disruptors [78,79,80] under helicase deficiency, for example, during HBV infection. Second, helicase interaction-based methods enable more effective rG4 studies, including non-canonical and in cellulo forms.

#### 3.3.2. FMRP: rG4-Binding Protein Inhibiting Translation

The FMR1 locus encodes the Fragile X Mental Retardation Protein (FMRP), a neuronally enriched RNA-binding protein whose dysregulation through compromised transcription or translation leads to neurodevelopmental disorders, including Fragile X Syndrome (FXS) and Fragile X-associated tremor/ataxia syndrome (FXTAS) [81,82]. The pathogenesis of these conditions is attributed to the dysregulation of FMRP-dependent gene expression networks, specifically perturbations in target mRNA translation and subcellular localization. FMRP’s molecular architecture includes three distinct RNA-binding modules: RGG, KH1, and KH2 domains. The crystallographic analysis revealed the RGG domain to recognize folded rG4s specifically, with protein binding occurring at the duplex–rG4 junction [83]. Biochemical analyses demonstrate that FMRP-rG4 interactions result in translational repression, as evidenced by luciferase-based reporter systems [84]. The mechanism appears to involve both rG4 stabilization and interference with ribosomal scanning. Additionally, FMRP mediates cap-dependent translational inhibition through CYFIP1 recruitment, which modulates eIF4E activity. While FMRP predominantly associates with rG4s within CDS and 3′-UTRs—including an autoregulatory interaction within its own coding region—at least three characterized mRNAs are regulated through 5′-UTR rG4 interactions.

**Figure 4:**
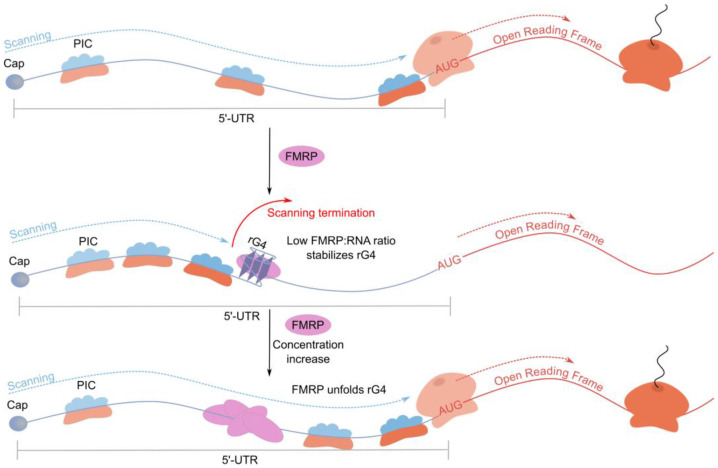
FMRP regulates the translation of MAP1B mRNA through rG4 in the 5′-UTR.

A notable example is microtubule-associated protein 1B (MAP1B), whose expression FMRP downregulates during developmental synaptogenesis. In FMR1-deficient conditions or knockout models, MAP1B overexpression leads to aberrant microtubule stabilization and impaired dendritic spine development [65]. Structural and biochemical characterization of the MAP1B 5′-UTR revealed a G-rich sequence capable of forming rG4, confirmed through CD and NMR spectroscopy [85]. The RGG domain of FMRP exhibits nanomolar affinity for this structure, primarily through hydrophobic interactions. Notably, the FMRP:mRNA stoichiometry influences rG4 stability: low ratios promote structure formation while higher ratios induce destabilization, suggesting a potential activity-dependent translational control mechanism during neuronal stimulation (Figure 4).

Studies have demonstrated that FMRP functions as a translational repressor of protein phosphatase 2A catalytic subunit (PP2Ac) mRNA [86]. In FMRP-deficient conditions, elevated PP2Ac expression disrupts cytoskeletal dynamics, specifically affecting dendritic spine morphogenesis in FXS patients. Biochemical analyses have revealed that FMRP exhibits nanomolar binding affinity for the PP2Ac 5′-UTR mRNA, which harbors four distinct rG4s—two exhibiting high stability and two with lower one, as determined by RT-stop assay. Detailed structural characterization of one particular rG4, positioned 4 nt upstream of the translation initiation site, was accomplished through CD spectroscopy and RNase T1 footprinting, enabling precise identification of nucleotides involved in rG4 formation [87]. Functional analyses using DLR assay demonstrated this rG4’s capacity to suppress translation by 70% relative to its non-rG4-forming mutated form.

Further investigations identified an additional rG4-forming sequence at position 163 within the 333 nt 5′-UTR of SMNDC1 mRNA [88]. SMNDC1, a splicing regulatory factor expressed in muscle tissue and neural structures, whose elevated expression correlates with apoptotic activation, was identified as a potential FMRP target through PAR-CLIP analysis. Biophysical characterization using CD spectroscopy, NMR analysis, and UV melting studies confirmed the formation of a three-tetrad rG4, which is stable at physiological conditions. EMSA demonstrated high-affinity, selective binding of FMRP’s RGG domain to this rG4, which was corroborated by RNA pull-down experiments. While direct evidence for rG4-mediated translational regulation of SMNDC1 by FMRP remains to be established, the observed interactions suggest a potential regulatory mechanism.

Additionally, while a rG4-forming sequence was identified in hASH1 mRNA 5′-UTR, comprehensive analysis using multiple experimental approaches revealed that a U10 uridine tract, rather than the rG4, serves as the primary regulatory element [89].

The accumulated experimental evidence indicates that rG4s exhibit regulatory capacities in translational control not only through creating structural impediments to scanning PIC but also by serving as recognition elements for regulatory trans-acting factors, particularly exemplified by FMRP-mediated translation regulation. However, comprehensive mechanistic investigations, employing methodological approaches similar to those utilized in hASH1 mRNA characterization, are required to establish detailed molecular mechanisms underlying rG4-dependent FMRP-mediated translational control in the identified regulatory systems.

## 4. rG4s Inhibiting Main ORF Translation Through Interaction with uORF

### 4.1. Synergistic Translation Inhibition by rG4s and Upstream Open Reading Frames

The inhibitory effect of rG4s on translation has been studied repeatedly at the transcriptome-wide level. A recent study focused on the relationship between upstream open reading frames (uORFs) and rG4s in inhibiting main ORF translation [90].

**Figure 5:**
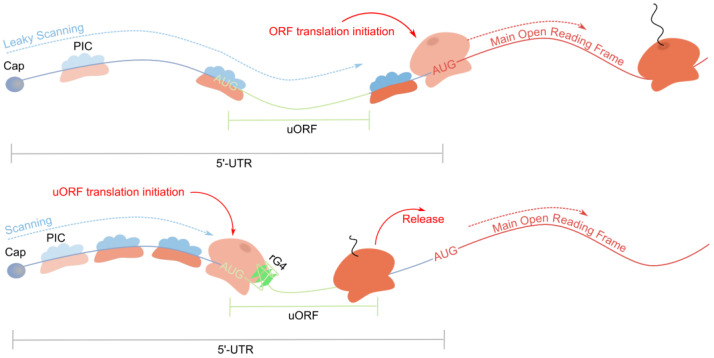
rG4s and upstream open reading frames synergistically inhibit translation of the main ORF.

Transcriptome-wide ribosomal profiling of cells treated with a translation elongation inhibitor (cycloheximide) was coupled with matched transcriptional mRNA sequencing and the calculation of length-corrected minimum free energies of folded RNA secondary structures in 5′-UTRs to observe the relationship between translation efficiency and secondary structure stability. Among mRNAs with rG4-regulated translation, the presence of 80S ribosome in 5′-UTR was observed more frequently than average, while less frequently in CDS, which indicated a more active uORF translation. The distribution of ribosomal footprints was non-random: (1) more actively translated uORFs corresponded to more stable rG4s; (2) rG4s were positioned downstream of uORF start codons in a way that generated queues of periodically scanning ribosomes. rG4s located downstream of uORF start codons force PICs to remain longer in the uORF start codon region, thereby increasing the probability of uORF translation initiation while inhibiting main ORF translation (Figure 5).

In the same study [90], the role of rG4-unwinding helicases in regulating the translation of mRNAs containing uORF and downstream rG4 was investigated. Polysome profiling coupled with proteomics mass spectrometry was employed. Data showed DHX9 and DHX36 helicases to concentrate in the polysomal fraction, so their mRNA binding corresponds to active translation. Ribosome profiling was subsequently repeated on cells with DHX9 and DHX36 knockdown. This revealed an additional increase and a decrease in uORF and the main ORF translation efficiency, respectively. Such mRNAs also contain more stable rG4s. The most critical factor in ensuring synergistic inhibition of main ORF translation through uORF–rG4 interaction is the position of rG4 relative to the uORF start codon (approximately 40 nt downstream) [90]. Translation efficiency changes are correlated for both helicases, indicating a shared target pool. Analysis of helicase-dependent translation revealed significant enrichment of genes encoding of transcription factors (e.g., STAT6 or FOXM1), epigenetic regulators (e.g., SUZ12, MLL1, or MLL5), and kinases (e.g., MAPK3, MAP2K1, or CDC42BPB). Individual-nucleotide resolution UV crosslinking and the immunoprecipitation (iCLIP) method were used to map DHX9 binding sites to determine whether DHX9 binds mRNAs directly via rG4s. The distribution of ribosomes on transcripts with DHX9 iCLIP peaks in 5′-UTRs showed enrichment of ribosome-protected fragments upstream of rG4-containing DHX9 binding sites in 5′-UTRs (though DHX9 was specified to process different secondary structures with a preference for RNA substrates) [90]. When DHX9 was depleted, ribosome occupancy in the 5′-UTR increased, while it decreased in the downstream coding sequence. These findings indicate that DHX9 regulates ribosome distribution and translation efficiency by directly binding to its rG4 substrate.

To further verify the roles of DHX9/DHX36 and their targets, GFP expressing vectors with 5′-UTRs containing either an rG4, a short rG4-containing uORF, or a non-rG4-forming mutant/alternative translation initiation site were constructed, using DDX23 5′-UTR as a template. The rG4 from DDX23 5′-UTR mRNA is characterized as being bound in cells by DHX9 and controlling its translation efficiency in a DHX36- and DHX9-dependent manner. Comparing GFP expression with an rG4 versus the non-rG4-forming mutant revealed minimal impact from the rG4 itself. Further tests showed that an alternative translation initiation site moderately influences expression. Interestingly, comparing the expression of the reporter gene with a 5′-UTR containing an alternative translation initiation site and a downstream rG4 to one containing a non-rG4-forming mutant revealed that the rG4 enhances the uORF’s repressive effect. Furthermore, the depletion of DHX36 or DHX9 resulted in a reduction in GFP expression in the context of an rG4-containing uORF, whereas no effect was observed in the analog with a mutated rG4. The data demonstrated that the repressive effect of the uORF was DHX36- and DHX9-dependent, and observed only in the presence of rG4. Thus, this study discovered and showed synergy between uORF and downstream rG4 in inhibiting main ORF translation.

### 4.2. Repeat-Associated Non-AUG Translation

**Figure 6:**
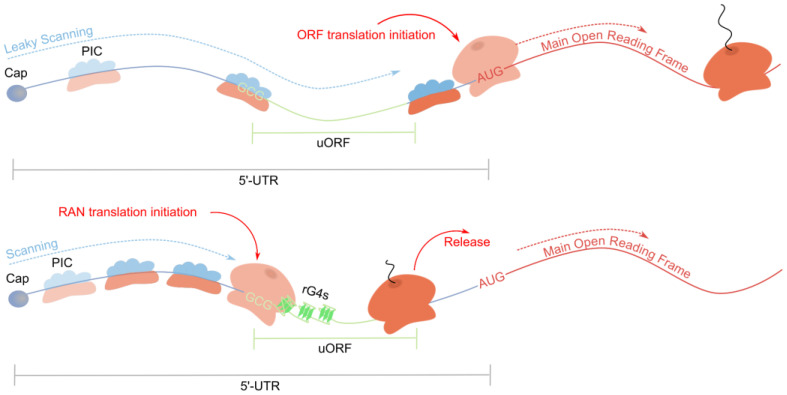
rG4s promote repeat-associated non-AUG translation.

Secondary structures can also cause translation from non-AUG start codons and inhibit cap-dependent translation of the main ORF [91]. This is especially characteristic of microsatellite repeats associated with the development of neurodegenerative disease and the synthesis of aggregation-prone toxic peptides and is called repeat-associated non-AUG (RAN) translation (Figure 6) [92]. A proposed mechanism for amyotrophic lateral sclerosis (ALS) and frontotemporal dementia (FTD) is the RAN translation of the r(G_4_C_2_)_n_ hexanucleotide repeat in C9Orf72 pre-mRNA, causing accumulation of toxic polyPR/polyGR dipeptide repeats [92,93]. An additional example is FXTAS, where affected individuals possess 55–200 repeats of the r(GGC)_n_ sequence in FMR1 mRNA 5′-UTR, leading to the production of toxic polyG, polyR, and polyA, as well as chimeric peptides [73,74]. Importantly, when 200–2000 repeats accumulate, transcript production halts due to methylation of CpG islands in this GC-rich region, resulting in FXS, which is also associated with intellectual disability from FMR1 protein deficiency.

The inhibition of translation from the main ORF and initiation of non-canonical RAN translation likely result from the formation of a highly structured RNA region due to repeat expansion [92]. The PIC assembles on the cap structure and scans the 5′-UTR for a start codon, but secondary structures encountered during this process impede PIC movement, reducing translation efficiency. This slowdown increases the chances of near-cognate non-AUG codons being recognized as start codons, thereby triggering abnormal repeat translation [92]. Specifically, the mechanism is enhanced by the displacement of eIF1 protein from PIC by eIF5, which is crucial for accurate start codon recognition and maintaining PIC’s open scanning conformation [91]. Additionally, these structures can induce a frameshift in the repeat region after non-canonical translation begins, resulting in chimeric peptides that are more toxic and aggregation-prone compared to their homopeptide equivalents [94]. RAN translation also leads to sequestration of ribosomes and translation factors, necessitating specific corrective enzymes for their release.

The r(GGC)_n_ and r(G_4_C_2_)_n_ repeats can form both hairpins and rG4s, but there is no consensus in the literature about which secondary structure serves as a more relevant biological target for regulating translation in ALS, FTD, and FXTAS [95,96,97,98]. Proteins such as hnRNP A2 and CBF-A, which can bind RNAs and unwind rG4s, have been shown to alleviate the inhibitory effect of FMR1’s structured 5′-UTR on main ORF translation, though these rG4-unwinding proteins lack structural specificity [96]. The rG4-destabilizing ligand TMPyP4 alleviated translation inhibition [98]. Replacing the r(GGC)_n_ motif with r(GGN)_n_ still allows polyG production but inhibits hairpin formation without negatively affecting rG4 assembly, resulting in a notable reduction in peptides’ toxicity and ribosome trapping [93,94]. This suggests that hairpin structures contribute significantly to the pathological effects of RAN translation. As far as r(G_4_C_2_)_n_ from C9Orf72 is concerned, support for the hairpin theory arises from several observations: (1) the hairpin form is more kinetically favored and becomes more preferable as repeat length increases; (2) RAN translation is less efficient in samples folded in potassium-rich buffer favoring rG4 stabilization; (3) the impact of G4 ligands on RAN translation is less pronounced than that of hairpin-interacting agents [95]. Furthermore, the inherent complexity of these repetitive sequences complicates the determination of their precise structure and whether that structure is consistent throughout the sequence, making it challenging to fully comprehend the potential implications of their three-dimensional conformation and dynamic variations. Ultimately, while establishing secondary structures of GC-rich repeats remains uncertain, this interplay between canonical and RAN translation exemplifies secondary structures’ regulatory role, offering promising avenues for therapeutic applications in neurodegenerative diseases.

## 5. rG4s Enhancing Translation

### 5.1. rG4s Activating Translation as IRES Components

**Figure 7:**
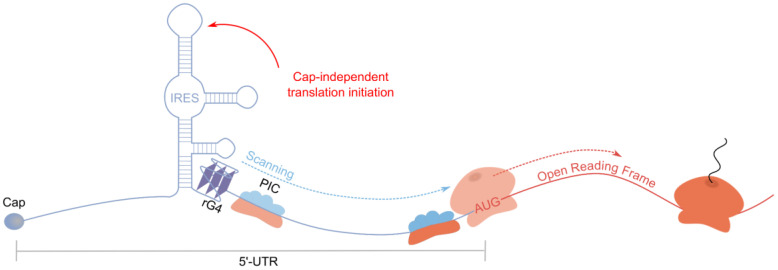
rG4s as a part of IRES promote cap-independent translation.

Until now, the role of rG4s in the regulation of cap-dependent translation has been discussed. However, rG4s can also mediate cap-independent translation, acting as elements of internal ribosome entry sites (IRESs) (Figure 7) [2,4]. The spatial structure of IRES allows for the recruitment of the 40S ribosomal subunit, an incomplete set of translation initiation factors, and IRES trans-acting factors (ITAFs) for translation initiation without the need for cap recognition [99]. The function of some smaller IRES elements, although lacking rG4, in eukaryotic cells has been described as mediating interactions with 18S rRNA in a Shine–Dalgarno-like manner, which is actually known in prokaryotes [100]. The subsequent investigation demonstrated that rG4s are able to directly interact with the 40S ribosomal subunit in the absence of other protein factors [101]. Most frequently, rG4s in the IRES region simultaneously inhibit cap-dependent translation and activate cap-independent translation, the engagement of which becomes necessary under stress conditions during the repression of canonical translation [20,62,102,103].

The first rG4, as an IRES component that enhances cap-independent translation efficiency, was found in the FGF2 mRNA transcript [37]. FGF-2 protein is involved in cell growth, migration, and differentiation, and its dysfunction is linked to diseases like cardiovascular disorders, osteoporosis, and neurodegenerative conditions [104]. The IRES controlling translation from four out of five start codons is localized in the first 176 nt of FGF2 mRNA 5′-UTR and contains two hairpins and a rG4 between them. Interestingly, rG4 represents an antiparallel five-tetrad chair-type rG4 with unusual two long loops, according to RT stop assay data. Since the main IRES element is the second hairpin’s loop, rG4 plays a rather supporting role and cannot by itself ensure efficient cap-independent translation, providing no more than 20% of IRES activity.

The second known rG4 activating translation was discovered in the IRES-A of VEGF mRNA 5′-UTR. Despite VEGF rG4’s widespread recognition, its role in translation regulation remains unclear. Human VEGF is a key angiogenic growth factor. In 2010, a G-rich sequence capable of forming a wide array of two-tetrad rG4s was shown to exist in VEGF mRNA IRES-A [105]. The introduction of four G-U mutations that prevent rG4 formation almost completely eliminated IRES-A activity, suggesting that rG4 was absolutely necessary for IRES-A-mediated cap-independent translation. Through various combinations of two G-U mutations limiting possible rG4 arrangements, translation efficiency could be either significantly decreased, or slightly increased. This led to the concept of a switchable rG4 motif that could provide fine-tuning of cap-independent translation efficiency through adopting different rG4 structures. Later, another research group showed that the 40S ribosomal subunit has a high affinity for this G-rich motif, enabling the IRES-A function, although whether the effect was structure- or sequence-specific was not specified [101]. While four G-U mutations did reduce translation efficiency, G-A and G-C mutations at the same positions, despite preventing rG4 formation, resulted in translation efficiency nearly identical to the WT sequence [106]. The insertion of a more stable three-tetrad rG4 from NRAS mRNA 5′-UTR and the treatment of the WT system with G4 stabilizing ligands decreased cap-independent translation efficiency. Moreover, exposure of cells to G4-stabilizing ligand Phen-DC6 decreased the ratio of polysomal to non-polysomal VEGF mRNA, further supporting rG4’s inhibitory role in IRES-dependent VEGF mRNA translation. Importantly, G-U mutations introduced an AUG codon absent in WT mRNA, creating a uORF that could explain decreased translation efficiency upon rG4 disruption [101,105,106]. However, mutants with two and four G-U mutations both contained this AUG codon, but only the second caused decreased translation efficiency, while the first had no effect [105]. This might indicate that neither rG4 disruption nor uORF appearance caused translation efficiency reduction with G-U mutations. In summary, (1) rG4 is not necessary for VEGF IRES-A activity, (2) rG4 inhibits VEGF translation when stabilized by G4 ligands and may represent a therapeutic target, and (3) the reasons for the decreased translation efficiency with four G-U mutations remain unknown, but it’s determination could illuminate IRES-A functioning. Later, a new IRES-A activity regulation mechanism was discovered, A856 methylation, which could deactivate endogenous uORF start codon in IRES-A and recruit the YTHDC2/eIF4GI complex to trigger cap-independent translation [107]. Interestingly, the knockdown of METTL3 encoding N6-adenosine-methyltransferase 70 kDa subunit reduced the effect of the G4 stabilizer 360A, and the treatment with 360A decreased METTL3 knockdown’s impact, though both effects remained statistically significant. A connection between G-rich region mutations and A856 methylation might be worth investigating to establish the mechanism of VEGF cap-independent translation regulation through IRES-A.

BAG-1 mRNA translation regulation is particularly interesting. As mentioned earlier, BAG-1 5′-UTR rG4 mutation led to a 2.4–4.0-fold increase in cap-dependent translation efficiency depending on a cell line in DLR assays. However, BAG-1 translation regulation is quite complex: in addition to cap-proximal rG4, the 5′-end contains four start codons, including the first non-canonical CUG, as well as IRES before the third codon and uORF [102]. The first three start codons encode protein isoforms differing in N-terminal length and intracellular localization. DLR assay established that rG4 inhibits the translation of all three protein isoforms and uORF, exerting a general suppression of cap-dependent translation. The inhibitory effect intensified when cells were treated with G4 stabilizers cPDS and Phen-DC3. Despite the proximity to cap structure, the effect is not related to the disruption of cap-dependent synthesis or loss of affinity to the translation initiation factor eIF4E. Meanwhile, cap-independent translation showed a 20% decrease in efficiency when the rG4-forming sequence was mutated to prevent its folding. After unveiling the spatial structure of 5′-UTR with intact rG4 or non-rG4-forming mutant using the SHAPE method, IRES with mutated rG4 was shown to adopt a more rigid structure and remains in the closed conformation, presumably preventing interaction with previously established ITAFs, PTB-1, and PCBP1, thereby inhibiting translation. Thus, BAG-1 provides an excellent example of rG4’s distal influence on IRES functionality, unlike most cases where rG4 is its constituent element.

Similarly, rG4 in the 5′-UTR of actin-related protein 2 (ARPC2) mRNA regulates translation. ARPC2 is the part of the ARP2/3 protein complex participating in actin cytoskeleton branching and facilitating cell migration. The rG4 inhibits cap-dependent translation by 40%, according to DLR assay, and can bind to ribosomal proteins within the 43S pre-initiation complex [20]. The complex formation with rG4, rather than steric blocking alone, might be responsible for translation inhibition. Nevertheless, ARPC2 mRNA can also be translated cap-independently due to IRES presence. The study established its structure, with rG4 positioned in a hairpin loop within a cruciform structure [103]. Mutational destabilization of the rG4 resulted in a 30% reduction in cap-independent translation efficiency. However, determining the exact cause of this effect proved challenging: it could be due to either IRES structure changes or the inadvertent creation of new ITAF binding sites through mutations. Notably, ARPC2 cap-independent translation efficiency increases under stress conditions at high cell density, suggesting a possible connection between rG4-mediated regulation and cellular stress.

There exists another example of rG4 participation in cap-independent translation with its increasing influence under stress conditions. NRF2 functions as a key transcription factor that induces antioxidant and detoxification gene expression, providing cytoprotection to multiple organ systems [108]. Its translation efficiency increases under oxidative stress conditions, as demonstrated during the H_2_O_2_ treatment, both at the endogenous protein level and in a bicistronic reporter system. A rG4-forming sequence was identified in the 5′-UTR, and rG4 folding was confirmed by CD and NMR spectroscopy as well as DMS footprinting. The reporter system showed that the non-rG4-forming mutation eliminated the response to H_2_O_2_ treatment, indicating its important regulatory role in oxidative stress response. Liquid chromatography-tandem mass spectrometry (LC-MS/MS)-based proteomics was employed to identify related protein factors and revealed the rG4-interacting protein, eukaryotic elongation factor 1 alpha (EF1a). An increase in translation efficiency during the H_2_O_2_ treatment of the cells did not occur upon EF1a knockdown. Additionally, RIP and RNA pull-down methods showed that the association of EF1a with NRF2 rG4 increases during the H_2_O_2_ treatment. Thus, the case represents an example of stress-activated rG4-mediated translation and, importantly, revealed one of the rG4-interacting proteins as a translation regulator.

There are reports on rG4 targets in alpha-synuclein (SNCA) mRNA 5′-UTR [109,110]. SNCA is a neuronal protein primarily located in presynaptic terminals, which is involved in modulating synaptic neurotransmission, and implicated in the pathogenesis of Parkinson’s disease (PD) and other synucleinopathies. However, therapeutic targeting prospects for these rG4s are questionable. For cap-dependent translation, the mutation of only one of the three rG4 led to a 36% increase in protein levels without changes in mRNA level, but its formation was unlikely based on the sequence and was not confirmed in cuvette. Cap-independent translation efficiency decreased by less than 15% when mutating all three rG4-forming sequences, though the effect remained statistically significant. These data question rG4’s significance in SNCA mRNA 5′-UTR.

Thus, some mRNAs can be translated both canonically and cap-independently under stress conditions. rG4s in such mRNAs may serve dual roles, inhibiting cap-dependent translation while activating the cap-independent one, supporting the idea of the particular importance of rG4-mediated translation regulation under stress conditions. rG4s can either be part of the IRES structure or regulate its structure from a distance.

### 5.2. Other Translation-Enhancing rG4s

Previously, we discussed rG4s activating translation as IRES components. However, there are systems with rG4 translation activators functioning independently of IRES or lacking data on cap-independent translation. For instance, rG4 presence in FOXE3 mRNA 5′-UTR is known to enhance translation efficiency by 60% compared to non-rG4-forming mutant according to DLR data [55]. FOXE3 is a transcription factor that plays an important role in vertebrate lens formation.

rG4s in transforming growth factor-beta 2 (TGFβ2) mRNA 5′-UTR have been studied more thoroughly [111,112]. Two rG4s were discovered that jointly enhance translation efficiency, but there is no evidence that they are IRES elements. TGFβ2 is a multifunctional cytokine involved in embryogenesis, tissue repair, and immune response through regulation of cell proliferation, differentiation, and migration. It is crucial for heart development and linked to carcinogenesis. The first rG4 is located 313 nt downstream of the cap structure [111]. Although incorporating the rG4-forming sequence alone into 5′-UTR of a reporter system showed a decrease in translation efficiency compared to the non-rG4-forming mutant, it enhances translation efficiency by up to 2-fold compared to the non-rG4-forming mutant in the full-length TGFβ2 5′-UTR context. Subsequently, a second rG4-forming sequence located 138 nt downstream of the cap structure was discovered [112]. According to DLR data, compared to the double mutant unable to form both rG4s, the WT 5′-UTR sequence provides translation efficiency enhancement up to 224%, while the mutation of either rG4 reduces activation level to approximately 130%. Interestingly, through mutations in the loops of rG4 138 nt downstream of the cap structure, it was demonstrated that the G138–G155 regulatory element is active due to structure rather than sequence. The system of two translation-activating rG4s in TGFβ2 mRNA 5′-UTR is unique and deserves special attention due to the therapeutic potential of the target protein.

One more example of an IRES-independent rG4 translation activator is the rG4 in cellular inhibitor of apoptosis protein 1 (cIAP1) mRNA 5′-UTR [46]. cIAP1 modulates various signal transduction pathways, especially TNFα-mediated signaling, to promote cell survival. In its longest version, the 5′-UTR is 1.7 kb long and contains several regulatory elements, including stress-activated IRES and uORF. In addition to these elements, a highly conserved G-rich sequence containing seven G-tracts was discovered near the cap structure. Notably, the search was conducted using the G4RNA screener program, which utilizes machine learning results rather than specific rG4 motifs [22]. In cellulo rG4 formation was supported by the G4-RNA specific precipitation (G4RP) method. This involved the stabilization of transiently formed rG4 structures by using the G4 stabilizer BRACO-19, followed by formaldehyde cross-linking before cell lysis and the pull-down of rG4-forming RNA transcripts from the lysate using a biotin-linked biomimetic G4 ligand BioCyTASQ. Using luciferase reporter plasmids, mutation that is unable to form rG4 in the context of the entire 5′-UTR was shown to unexpectedly lead to a dramatic decrease in translation efficiency to a greater degree than typically observed for rG4s. When checking for possible rG4 influence on distal IRES, IRES activity levels under normal growth conditions proved insignificant and did not explain rG4’s activating effect. Thus, rG4 in cIAP1 mRNA 5′-UTR represents a unique example of a highly efficient and IRES-independent rG4 translation activator.

## 6. Discussion

Contemporary research findings indicate that rG4s facilitate complex translational control mechanisms. They can inhibit translation either through sterically hindering the PIC scanning of 5′-UTR or by interacting with proteins like FMRP. While inhibiting downstream main ORF translation, they can simultaneously activate translation from uORF and upstream near-cognate codons, thus acting as translation activators for these elements. In the case of rG4 located downstream of uORF, a synergistic inhibitory effect is observed. Moreover, helicases contribute significantly to regulating rG4-controlled translation. However, rG4s can activate translation: they can be integral parts of IRES, distally influence its structure, or even activate translation independently of it. In the latter case, mechanisms are insufficiently studied, though, in cIAP1’s case, it might be related to the PIC assembly efficiency due to cap-proximal positioning. Interestingly, in prokaryotes, rG4s in 5′-UTR have been shown to enhance main ORF translation efficiency [113]. Thus, the translation of rG4-containing mRNAs can be finely tuned and tightly regulated for rapid cell response to changing environmental conditions.

However, the field of rG4 influence study faces numerous methodological challenges and artifacts. As discussed above, the identification of rG4-forming sequences was traditionally limited to the canonical motif pattern. Further studies revealed the widespread occurrence of non-canonical rG4 structures, highlighting the need for new search methods. Contemporary methods can be generally divided into two groups: those using hand-crafted rules and those employing machine learning [114]. Among the former, G4Catchall and G4Hunter are the most effective, both allowing for the identification of a wide range of non-canonical motifs [24,32,114]. The G4RNA screener, used in cIAP1 and BAG-1 studies, was one of the first machine learning-based methods [22]. Recently, the G4Boost algorithm was developed, surpassing known machine learning methods in accuracy and providing a G4 thermodynamic stability assessment [34]. Thus, the toolkit for addressing this challenge has significantly improved.

The second methodological challenge derives from the usage of reporter systems as primary methods for confirming rG4s’ influence on translation. Despite their widespread application and several undisputed advantages, such as rapid execution and the ability to provide precise quantitative results, they have several inherent limitations. The main limitations are inadequate corresponding mRNA levels and increased cellular process load due to viral promoter usage [52,115,116]. This substantially alters the distribution and accessibility of enzymes involved in transcription and translation. This alone causes cross-influence between test and normalization plasmid expression and reduces endogenous gene expression levels, including housekeeping genes [115,116]. Thus, the test does not adequately model the effects of regulatory elements during endogenous expression. Additionally, the results strongly depend on experimental conditions: chosen promoter, transfection efficiency, and plasmid quantity, making data comparison challenging. Therefore, the application of developed robust methods to study rG4 effects at endogenous mRNA levels, such as genome editing and helicase knockdown, is necessary.

The third methodological challenge involves the accurate sequencing of mRNA cap-proximal regions. It is hindered by the lack of transcription start site-specific signal sequence and the presence of transcriptional noise [117,118]. Moreover, RNA-seq read lengths are typically shorter than those required for high-quality mapping, and standard RNA-seq does not prevent the production of 5′-truncated sequences. To address this, methods were developed using special primers for 5′-end amplification (5′-RACE) or cap-containing mRNA pull-down (CAGE) [119]. The problem is that these methods were developed significantly later than the first studies on rG4-mediated translation regulation were published, so revalidations of well-known rG4 targets are urgently required. Annotation and mRNA sequence refinement according to new data continues to this day. Moreover, transcription start sites and splicing can vary depending on cell line and condition, producing different mRNA sets. These challenges explain why the NRAS rG4, discovered in 2007 and becoming a classic example, was not detected in at least 14 cell lines in 2024 [58].

Contemporary rG4 research necessitates both the development of novel methodologies for evaluating endogenous rG4-mediated effects and the systematic reevaluation of previously identified rG4 targets using advanced analytical techniques, especially to confirm their presence in cellular mRNAs.

## Figures and Tables

**Table 1:** The 5′-UTR rG4s inhibiting translation.

Target	Function	Methods Used for Verification of rG4s Effect on Translation	rG4’s Effect on Translation, Fold Change Versus Mutant	Ref.
*NRAS*	Membrane protein regulating cell proliferation and differentiation, associated with cancers	UV-melting (Tm = 63 °C at 1 mM KCl)/in vitro translation	3.6 ^1^	[42]
*ZIC-1*	Transcription factor regulating cerebellar development, overexpressed in medulloblastoma	UV-melting (Tm = 79 °C at 25 mM KCl)/DLR, Western blotting	5 ^2^	[43]
*ESR1*	Transcription factor, regulating cell proliferation and differentiation, upregulated in estrogen receptor-positive breast cancers	CD-melting (Tm > 85 °C at 100 mM KCl)/in vitro translation	6 ^1^	[51]
*TRF2*	Component of the telomere nucleoprotein shelterin complex, overexpressed in cancers	UV-melting (Tm = 62 °C at 1 mM KCl)/Western blotting	2.45 ^3^	[44]
*CCND3*	Cyclin regulating the G0/G1 to S phase transition, overexpressed in cancers	CD-melting (Tm = 73 °C at 1 mM KCl), RNase T1/Western blotting	2 ^2^	[41]
*BCL-2*	Integral outer mitochondrial membrane protein that blocks apoptosis and associated with lymphomas	UV-melting (Tm = 81 °C at 50 mM KCl)/DLR, in vitro translation, Western blotting	3.5 ^1^, 1.4–1.9 ^2^	[39,52]
*ADAM10*	α-Secretase of a disintegrin and metalloproteases family with anti-amyloidogenic activity	CD-melting (Tm = 60 °C at 1 mM KCl)/DLR, in vitro translation, Western blotting	3 ^1,2^, 9–15 ^3^	[45]
*MT3-MMP*	Matrix metalloproteinase degrading extracellular matrix proteins, associated with the invasiveness of cancers	CD-melting (Tm = 72 °C at 1 mM KCl), RNase T1/DLR	2.2 ^2^	[53]
*EBAG9*	Activator of apoptotic protease, associated with cancers	in-line probing/DLR	1.8 ^2^	[54]
*FZD2*	Transmembrane receptor coupled to the beta-catenin canonical signaling pathway, associated with omodysplasia, Robinow syndrome and hepatocellular carcinoma	in-line probing/DLR	2.5 ^2^	[54]
*BARHL1*	Transcription factor involved in midbrain development/neuron migration, associated with medulloblastoma	in-line probing/DLR	1.9 ^2^	[54]
*NCAM2*	Membrane protein regulating cell adhesion/neuron adhesion, associated with epilepsy and familial temporal lobe	in-line probing/DLR	1.6 ^2^	[54]
*THRA*	Thyroid hormone receptor, associated with hypothyroidism	in-line probing/DLR	1.6 ^2^	[54]
*AASDHPPT*	Phosphopantetheinyl transferase, associated with pipecolic acidemia and lissencephaly	in-line probing/DLR	2.2 ^2^	[54]
*AKTIP*	Protein interacting with protein kinase B (PKB)/Akt and modulating its activity	UV-melting (Tm = 79 °C at 1 mM KCl)/DLR	1.3 ^2^	[55]
*CTSB*	Lysosomal cysteine proteinase involved in the proteolytic processing of amyloid precursor protein	UV-melting (Tm = 68 °C at 1 mM KCl)/DLR	1.4 ^2^	[55]

Effects on translation of rG4-forming sequence versus non-rG4-forming mutant(s) were measured by means of ^1^ in vitro translation, ^2^ DLR, ^3^ Western blotting.

## Data Availability

Not applicable.
